# Modeling of Frontotemporal Dementia Using iPSC Technology

**DOI:** 10.3390/ijms21155319

**Published:** 2020-07-27

**Authors:** Minchul Kim, Hee Jin Kim, Wonyoung Koh, Ling Li, Hyohoon Heo, Hanna Cho, Chul Hyoung Lyoo, Sang Won Seo, Eun-Joo Kim, Mahito Nakanishi, Duk L. Na, Jihwan Song

**Affiliations:** 1 Department of Biomedical Science, CHA Stem Cell Institute, CHA University, 335 Pangyo-ro, Bundang-gu, Seongnam-si, Gyeonggi-do 13488, Korea; mckim83@gmail.com (M.K.); wyoung0123@naver.com (W.K.); leeling182858@gmail.com (L.L.); hhhun111@naver.com (H.H.); 2 Neuroscience Center, Samsung Medical Center, 81 Irwon-ro, Gangnam-gu, Seoul 06351, Korea; evekhj@gmail.com (H.J.K.); sangwonseo@empal.com (S.W.S.); 3 Department of Neurology, Samsung Medical Center, Sungkyunkwan University School of Medicine, 81 Irwon-ro, Gangnam-gu, Seoul 06351, Korea; 4 Samsung Alzheimer Research Center, Samsung Medical Center, 81 Irwon-ro, Gangnam-gu, Seoul 06351, Korea; 5 Department of Health Sciences and Technology, SAIHST, Sungkyunkwan University, 81 Irwon-ro, Gangnam-gu, Seoul 06351, Korea; 6 Stem Cell & Regenerative Medicine Institute, Samsung Medical Center, 81 Irwon-ro, Gangnam-gu, Seoul 06351, Korea; 7 Department of Neurology, Gangnam Severance Hospital, Yonsei University College of Medicine, Seoul 06273, Korea; iguhanna@naver.com (H.C.); LYOOCHEL@yuhs.ac (C.H.L.); 8 Department of Neurology, Pusan National University Hospital, Pusan National University School of Medicine and Medical Research Institute, Busan 49241, Korea; eunjookim@pusan.ac.kr; 9 TOKIWA-Bio, Inc., Tsukuba Center Inc. (TCI), Building G, 2-1-6 Sengen, Tsukuba, Ibaraki 305-0047, Japan; nakanishi@tokiwa-bio.com; 10 iPS Bio, Inc., Rm 302-8, 26 Yatap-ro, Bundang-gu, Seongnam-si, Gyeonggi-do 13522, Korea

**Keywords:** frontotemporal dementia (FTD), induced pluripotent stem cells (iPSC), disease modeling, cell death, staurosporine (STS), drug screening

## Abstract

Frontotemporal dementia (FTD) is caused by the progressive degeneration of the frontal and temporal lobes of the brain. Behavioral variant FTD (bvFTD) is the most common clinical subtype of FTD and pathological subtypes of bvFTD are known as FTD-tau, transactive response (TAR) DNA-binding protein 43 (TDP-43), and fused in sarcoma (FUS). Pathological mechanisms of bvFTD are largely unknown. In this study, we investigated the expression of pathological markers, such as p-Tau, TDP-43, and FUS, in the induced pluripotent stem-cell-derived neurons (iPSN) from two sporadic bvFTD patients and one normal subject. We also used an FTD-patient-derived iPSC-line-carrying microtubule-associated protein tau *(MAPT*) P301L point mutation as positive control for p-Tau expression. Staurosporine (STS) was used to induce cellular stress in order to investigate dynamic cellular responses related to the cell death pathway. As a result, the expression of active caspase-3 was highly increased in the bvFTD-iPSNs compared with control iPSNs in the STS-treated conditions. Other cell-death-related proteins, including Bcl-2-associated X protein (Bax)/Bcl-2 and cytochrome C, were also increased in the bvFTD-iPSNs. Moreover, we observed abnormal expression patterns of TDP-43 and FUS in the bvFTD-iPSNs compared with control iPSNs. We suggest that the iPSC technology might serve as a potential tool to demonstrate neurodegenerative phenotypes of bvFTD, which will be useful for studying pathological mechanisms for FTD as well as related drug screening in the future.

## 1. Introduction

Frontotemporal dementia (FTD) is the second-most common cause of neurodegenerative dementia, showing behavioral problems typically in people younger than 65 years of age [1,2]. Most importantly, it prominently features atrophy in the frontal and temporal lobes leading to frontotemporal lobar degeneration (FTLD) associated with a wide range of heterogeneous pathologies. In other words, it is characterized by selective death of cerebral cortex neurons [3].

Behavioral variant FTD (bvFTD) is the most common clinical subtype of FTD, and pathological subtypes of bvFTD are classified into FTD-tau, TAR DNA-binding protein 43 (TDP-43), and fused in sarcoma (FUS) [2,4,5,6]. Genetic mutations linked to FTD include microtubule-associated protein tau *(MAPT),* progranulin *(PGRN),* and chromosome 9 open reading frame 72 *(C9orf72)* [7], which have been targeted for studying pathological mechanism or discovering new treatments. Data from European ancestry revealed that the familial forms of FTD represent about 40–50% of FTD [2,6]. However, in the East Asia, familial FTD cases are extremely rare [8,9,10,11]. Clinical diagnosis of sporadic bvFTD relies on clinical diagnostic criteria [3]. However, there are still misguided cases in diagnosis, and the differential diagnosis of bvFTD just based on clinical symptoms has been challenging. Moreover, genetic and pathological mechanisms of sporadic bvFTD are largely unknown. More recently, Genome-wide association studies (GWAS) on various clinical FTD subtypes have been reported to better define their genetic architecture [6]. Therefore, it is important to unravel the pathological mechanisms of sporadic bvFTD as well as to identify suitable biomarkers, in order to establish more useful diagnostic guidelines and to discover new therapeutic targets.

Induced pluripotent stem cells (iPSC) have become a pivotal tool to recapitulate the disease phenotypes of patients [2]. Several studies involving the generation and characterization of familial FTD patient-derived iPSC, as well as familial Alzheimer’s disease patient-derived iPSC have been reported recently [12,13,14,15,16]. However, most of the FTD cases are sporadic especially in East Asia and iPSC generation and characterization from sporadic bvFTD is challenging.

In this study, we generated patient-specific iPSC lines from two young sporadic bvFTD patients using their peripheral blood mononuclear cells (PBMCs) and characterized their neurons (bvFTD-iPSNs) with respect to the expression level of FTD-tau, TDP-43, FUS, and activated caspase-3. Among them, immunocytochemical and immunoblot analyses indicated that the active caspase-3 expression, indicative of neurodegenerative feature, was significantly increased compared with controls. This neurodegenerative feature of FTD can be used as a useful marker for studying pathological mechanisms, as well as drug screening.

## 2. Results

### 2.1. Case Description

**Figure 1:**
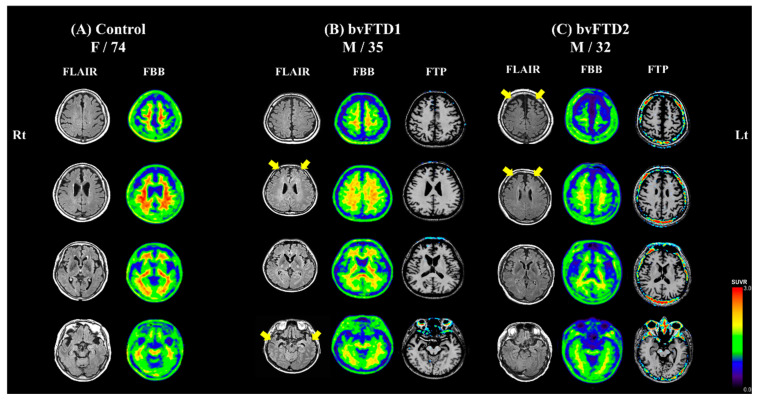
Brain images of participants. Axial views from top (in the first row) to bottom (in the last row) section of the brain are shown. (**A**) The 74-year-old normal control subject showed no atrophy on fluid-attenuated inversion recovery (FLAIR) image. Florbetaben (FBB) PET showed no abnormal uptake, indicating no significant amyloid-beta deposition. (**B**) The 35-year-old sporadic behavioral variant frontotemporal dementia (bvFTD) patient showed a marked atrophy in the fronto-temporal area on FLAIR image (yellow arrows). His FBB PET showed no abnormal uptake. Flortaucipir (FTP) PET showed no abnormal uptake, indicating no significant paired helical filament tau deposition. (**C**) The 32-year-old sporadic bvFTD patient showed a prominent atrophy in the frontal area on FLAIR image (yellow arrows). FBB PET and FTP PET showed no abnormal uptake.

The normal control subject, a 74-year-old woman, did not show cognitive impairment on neuropsychological testing. Her brain MRI showed no atrophy and florbetaben PET showed no abnormal uptake (Figure 1A).

The bvFTD1 patient, a 35-year-old previously-healthy man, visited Samsung Medical Center for two years of progressive disinhibition, apathy, compulsive behavior, and loss of empathy. He also showed utilization behavior and addiction to smoking. Neuropsychological testing [17] revealed frontal dysfunction relatively sparing of memory function. The bvFTD1 patient did not show parkinsonism or other movement problems. He did not have family history of dementia or other neuropsychiatric disorders. The patient’s MRI showed marked atrophy in the fronto-temporal area for his age. Thus, he met consensus diagnostic criteria for probable bvFTD [3]. Florbetaben PET showed no abnormal uptake, indicating no significant amyloid-beta deposition. Flortaucipir PET showed no abnormal uptake, indicating no significant paired helical filament tau deposition (Figure 1B).

The bvFTD2 patient, a 32-year-old previously-healthy man visited Samsung Medical Center for three years of progressive disinhibition, apathy, binge eating, and aphasia. On neuropsychological test [17], he showed frontal dysfunction including perseveration and transcortical motor aphasia. He did not show parkinsonism or other movement problems. The bvFTD2 patient’s maternal grandfather had dementia at the age of 60. However, his mother was still healthy and cognitively normal. The patient’s MRI showed prominent atrophy in the frontal area for his age. Thus, he met the consensus diagnostic criteria for probable bvFTD [3]. Florbetaben PET and flortaucipir PET showed no abnormal uptake. His cognitive decline and abnormal behavior worsened rapidly (Figure 1C). One year after his first visit, he became mute and his aggressive behavior was uncontrollable.

Whole-exome sequencing (WES) was performed using peripheral blood from the two FTD patients. Forty-six genes that are related to FTD, ALS, or other dementias were screened for pathogenic variants: *ALS2, ANG, CHMP2B, CHRNA4, DAO, DCTN1, FIG4, FUS, GRN, HNRNPA1, HNRNPA2B1, MAPT, MATR3, OPTN, PRNP, SETX, SIGMAR1, SOD1, SPG11, SQSTM1, TAF15, TARDBP, TBK1, TREM2, UBQLN2, VAPB, VCP, AARS2, ABCD1, APP, ARSA, CSF1R, DARS2, EIF2B1, EIF2B2, EIF2B3, EIF2B4, EIF2B5, GALC, GBA, GLA, NOTCH3, PSEN1, PSEN2, SNCB,* and *TYROBP.* We did not identify likely pathogenic variants or pathogenic variants in dementia-related genes in the two FTD patients.

In the bvFTD2 patient, *C9orf72* repeat expansion was tested but it turned out to be normal. In the case of bvFTD1 patient, we did not test for C9orf72 repeat expansion. However, unlike in Europe, no pathogenic GGGGCC repeats in C9orf72 were reported to cause FTD in East Asia [18,19,20].

### 2.2. Generation and Characterization of the bvFTD Patient-Derived iPSCs

**Figure 2:**
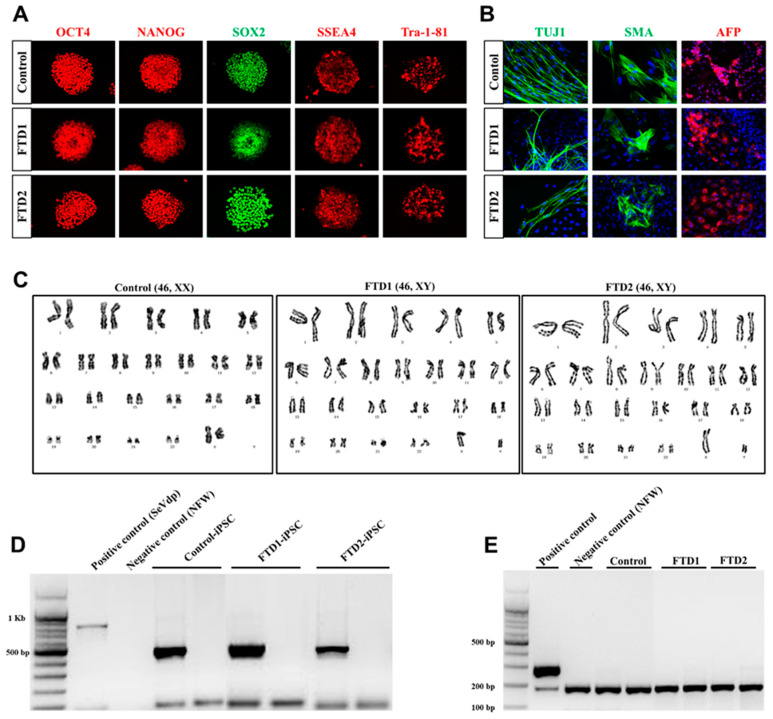
Generation and characterization of induced pluripotent stem cells (iPSCs) derived from bvFTD patients. (**A**) Expression of undifferentiated pluripotent stem cell markers in the established iPSC lines from control and two bvFTD patients. octamer-binding transcription factor-4 (OCT4, red), NANOG (red), SRY-box transcription factor 2 (SOX2, green), stage-specific embryonic antigen 4 (SSEA4, red) and tumor rejection antigen-1-81 (TRA-1-81, red). (**B**) Immunofluorescence analysis showing the differentiation potential of iPSC lines into three germ layers, including ectoderm type III β-tubulin (TUJ1, green), mesoderm smooth muscle actin (SMA, green), and endoderm α-fetoprotein (AFP, red). Scale bar: 100 µm. (**C**) Karyotype analysis of the control and bvFTD-patient-derived iPSC lines. (**D**) Sendai virus vector clearance was confirmed by reverse transcription polymerase chain reaction (RT-PCR) (**E**) Mycoplasma contamination was confirmed from genomic DNA using the PCR-based e-Myco^TM^ VALiD mycoplasma detection kit (iNtRON, Korea).

Isolated mononuclear cells (MNCs) from the peripheral blood of patients were reprogrammed using Sendai virus vector (SeVdp) which expresses four reprogramming factors (OCT3/4, SOX2, c-MYC, and KLF4) [21]. We generated iPSC lines from one normal control and two bvFTD patients who were confirmed to have no FTD-related gene mutations, such as *PGRN* and *MAPT*, in their genomes. We also confirmed the expression of undifferentiated pluripotent stem cell markers, including octamer-binding transcription factor 4 (OCT4), SRY-box transcription factor 2 (SOX2), NANOG, stage-specific embryonic antigen 4 (SSEA4), and tumor rejection antigen-1-81 (TRA-1-81). Using embryoid body formation, we confirmed three germ-layer formation, judged by the expression of neuron-specific class III b-tubulin (Tuj1, a marker for ectoderm), smooth muscle action (SMA, a marker for mesoderm), and alpha-fetoprotein (AFP, a marker for endoderm) (Figure 2A,B). We also confirmed that no abnormal chromosome karyotypes were observed in our iPSC lines (Figure 2C). Finally, we confirmed the absence of residual Sendai virus vectors and mycoplasma contamination in all our iPSC lines (Figure 2D,E). Taken together, these results indicate that our bvFTD patient-derived iPSC lines meet the general criteria of iPSC.

### 2.3. Neuronal Differentiation of bvFTD-Patients-Derived iPSCs

All iPSC lines were differentiated into post-mitotic neurons according to the procedures shown in the schematic diagram (Figure S1A). Additionally, we purchased an FTD-patient-derived iPSC-line-carrying *MAPT P301L* point mutation from the National Institute of Neurological Disorders and Stroke (NINDS) as a positive control. In brief, we induced neural precursor cells (NPCs) from iPSCs using the serum-free culture of embryoid body-like quick aggregation, also known as SFEBq, method [22]. Immunocytochemical analyses confirmed the formation of NPCs, judged by the expression of NESTIN, SOX2, and MUSASHI (Figure S1B). Afterwards, we further differentiated NPCs into mature neurons for three weeks and then treated them with staurosporine (STS) for 24 h to induce cellular stress. In this study, we adopted our well-established differentiation protocols for cortical neurons [23], in which high levels of COUP-TF-interacting protein 2 (CTIP2), T-box brain transcription factor 1 (TBR1) and choline acetyltransferase (ChAT)-positive cortical neurons were robustly formed at 10 weeks of neuronal differentiation. The current protocol, however, shortened the differentiation time from 10 to 3 weeks, for the purpose of using these cells for drug screening in the future. To facilitate the induction of neuronal pathology in the sporadic bvFTD-patient-derived iPSNs, we introduced the STS treatment to induce extrinsic cellular stress.

### 2.4. Cell Death Marker Expression in the bvFTD iPSC-Derived Neurons (iPSNs)

The most prominent feature of FTD is the cerebral atrophy, indicative of loss of neurons [24]. Previous studies have reported that the expression of various cell-death-related proteins is elevated in FTD-iPSNs carrying gene mutations, because disease progression ultimately leads to neuronal cell death [18,19,20]. For this reason, we first examined the expression of cell-death-related proteins in the bvFTD-iPSNs, together with control iPSC-derived neurons. To increase the difference, we treated these neurons with STS, a cell-death inducer, which inhibits kinase activity, thereby inducing cellular stress condition in the neuronal culture [13].

**Figure 3:**
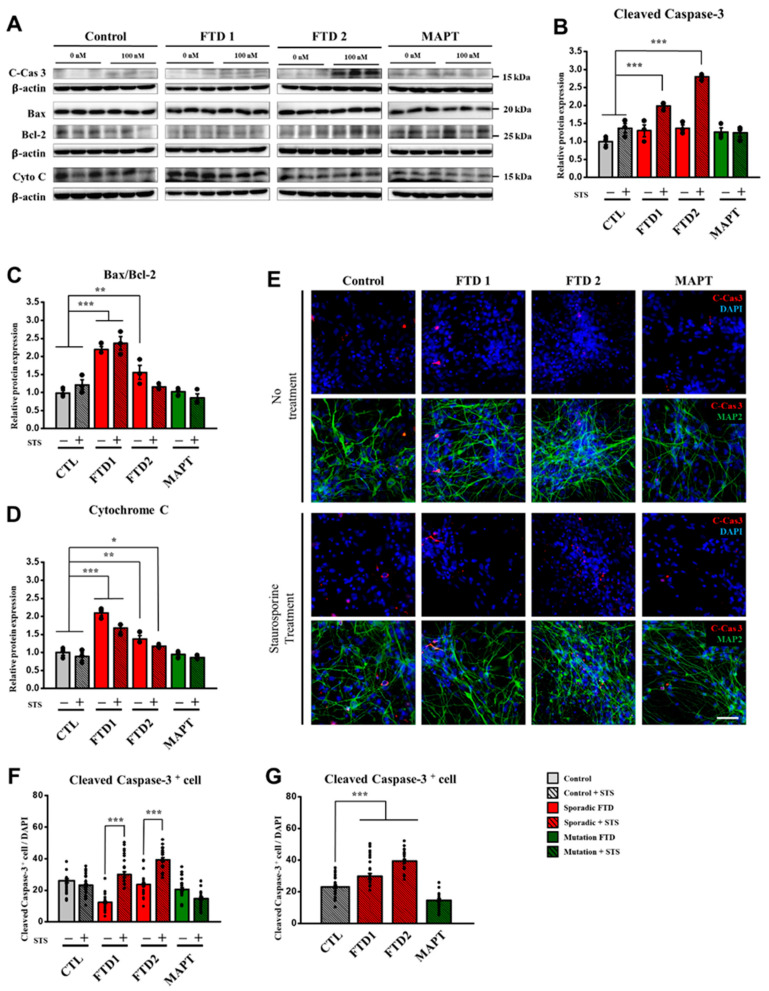
Expression patterns of cell-death-related proteins in the bvFTD iPSC-derived neurons in response to staurosporine (STS) treatment. (**A**) Immunoblot assay showing the expression of cell-death-related markers, including cleaved caspase-3 (17 kDa), Bax (21 kDa), Bcl-2 (26 kDa), and cytochrome C (14 kDa). Note that samples were obtained from two different culture conditions in the presence or absence of STS treatment (100 nM). (**B**) Quantification of immunoblot assay showing that cleaved caspase-3 expression was significantly increased in the two bvFTD-iPSNs compared with the normal control. (**C**) Quantification of immunoblot assay by obtaining the ratio of Bax and Bcl-2 expression showing that significantly-increased patterns were observed in the two bvFTD-iPSNs compared with the normal control. (**D**) Quantification of immunoblot assay showing that cytochrome C expression was significantly increased in the two bvFTD-iPSNs compared with the normal control. Note that blot images indicated by dividing lines were chosen from different immunoblots due to the large size of samples. Whole blot images are provided in Supplementary Figure S3A–D. Protein quantifications were conducted under the same exposure time (*n* = 3) (*: vs. normal control) * *p* < 0.05, ** *p* < 0.01, *** *p* < 0.001, one-way ANOVA. (**E**) Immunocytochemical assay showing the expression of cleaved caspase-3 (red) and MAP2 (green), which were counter-stained with 4′,6-diamidino-2-phenylindole (DAPI, blue) in all iPSC-derived neurons at three weeks of differentiation. Note that samples were obtained from two different culture conditions in the presence or absence of STS treatment (100 nM). Scale bar: 50 μm. (**F**) Quantification of immunocytochemical assay by using the co-localization module in the MetaXpress^®^ 6 program. Note that all the images quantified by the program were normalized by total number of nuclei (*: vs. each line without STS treatment) * *p* < 0.05, ** *p* < 0.01, and *** *p* < 0.001, one-way ANOVA. (**G**) all bvFTD-iPSNs showed significantly-increased active caspase-3 in the presence of STS treatment compared with the normal control. (*n* =36) (*: vs. normal control) * *p* < 0.05, ** *p* < 0.01, and *** *p* < 0.001, one-way ANOVA.

We first quantified the expression level of active caspase-3, a 16 kDa fragment after cleavage, from immunoblot assay. Interestingly, the expression of active caspase-3 was significantly increased in the bvFTD-iPSNs after STS treatment (Figure 3A,B). However, no significant difference was observed in the normal and positive controls after STS treatment (Figure 3A,B). Moreover, we measured the expression of cleaved caspase-3 around the nuclei in the soma area using the co-localization module of MetaXpress^®^ 6 program (Molecular Devices, San Jose, CA, USA). As a result, the two bvFTD-iPSNs exhibited significantly-increased expression of cleaved caspase-3 after STS treatment, compared with their untreated controls (Figure 3E,F) and the normal control (Figure 3G). Therefore, these results strongly suggest that bvFTD-iPSNs exhibit higher vulnerability to cell death under cellular stress condition, compared with controls.

By contrast, both immunoblot and immunocytochemistry results showed no significant differences in the expression level of cleaved caspase-3 between the normal control and positive control carrying *MAPT P301L* mutation (Figure 3B,G). These results strongly suggest bvFTD-iPSNs are highly vulnerable to cell death under cellular stress condition, and the level of cleaved caspase-3 can be utilized as a useful biomarker when these cells are used for drug screening.

To verify our findings, we also examined other cell-death-related markers, such as Bcl-2, Bax, and cytochrome C by the immunoblot assay (Figure 3A and Figure S3A–D). Interestingly, we observed that both Bax/Bcl-2 ratio and cytochrome C expression level were significantly increased in the two bvFTD-iPSNs, even without STS treatment, compared with the normal control (Figure 3C,D). Unlike our expectation, *MAPT P301L*-iPSN did not strongly react to the STS treatment, and no significant difference was observed, compared with the normal control. Given that no change of cell-death-related proteins involved in mitochondria permeability transition was induced by the STS treatment, it is likely that *MAPT P301L*-iPSN has mitochondrial resilience against the STS treatment (at 100 nM), compared with the two bvFTD-iPSNs. Taken together, these results further demonstrate that bvFTD-iPSNs are highly vulnerable to cell death, in the presence or absence of STS treatment.

### 2.5. Amyloid Beta Secretion and p-Tau Expression in the bvFTD iPSC-Derived Neurons

In both bvFTD patients, florbetaben PET and flortaucipir PET were all negative which indicated that the two patients did not have amyloid plaque or paired helical filament tau in the brain (Figure 1A). Therefore, we quantified amyloid beta peptide 42 (Aβ_42_) normalized with Aβ_40_ (amyloid beta peptide 40) from the conditioned medium, and we confirmed that bvFTD-iPSNs did not exhibit abnormal expression of amyloid peptides, compared with controls including familial Alzheimer’s disease (AD) iPSC-derived neuron (*PSEN1 S170F* mutation) that had been confirmed in our previous report (Figure S2) [23].

Since *MAPT* mutation causes familial FTD, we included the *MAPT P301L* cell line, which represents the FTD tauopathy model showing 4-repeat (4R) isoforms of tau, mitochondrial dysfunction, early maturation in neuronal cells, and eventually, cell death [25,26,27]. Therefore, we analyzed the expression of phosphorylated Tau (*p*-Tau), using antibody-targeting AT8 epitope (Ser202, Thr205) and Tau5 (Total Tau) by immunocytochemistry and immunoblot assays.

**Figure 4:**
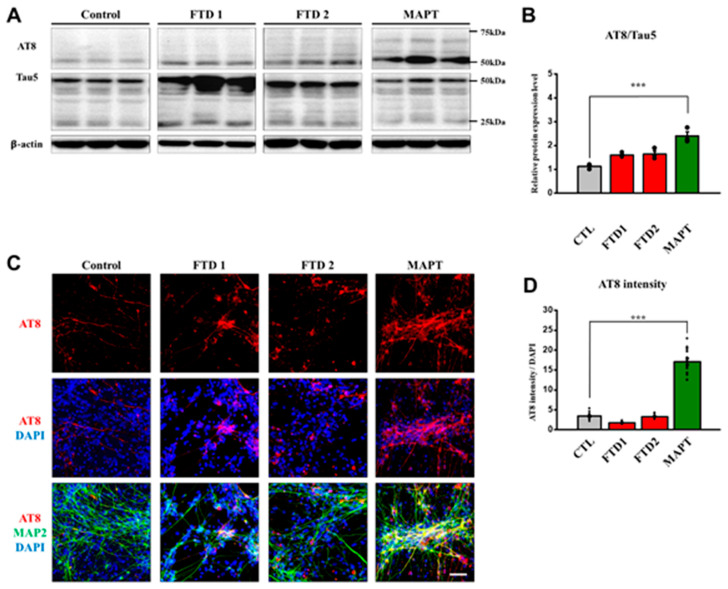
Characterization of phosphorylated tau expression pattern in the bvFTD iPSC-derived neurons. (**A**) Immunoblot assay showing a significant increase of phosphorylated tau (AT8), and total tau (Tau5) proteins in the FTD iPSC-derived neurons. Note that blot images indicated by dividing lines were chosen from different immunoblots due to the large size of samples. Protein quantifications were conducted under the same exposure time. Whole blot images are provided in Supplementary Figure S3E. (**B**) Quantification of AT8 expression against its own total tau (tau 5) expression in the iPSC-derived neurons (*n* = 3) (*: vs. normal control) * *p* < 0.05, ** *p* < 0.01, and *** *p* < 0.001, one-way ANOVA (**C**) Immunocytochemical assay showing the expression of AT8 (red) and MAP2 (green), which were counter-stained with DAPI (blue), in the iPSC-derived neurons at three weeks of neuronal differentiation. Scale bar: 100 μm. (**D**) Quantification of the intensity of AT8 protein expression with respect to MAP2-positive neurons, which were normalized against DAPI (*n* = 16) (*: vs. control) * *p* < 0.05, ** *p* < 0.01, and *** *p* < 0.001, one-way ANOVA.

First, we quantified AT8 and Tau5 expression by immunoblot assay. The hyperphosphorylated tau expression against total tau expression did not show significant differences between the normal control and bvFTD-iPSNs. By contrast, *MAPT P301L* cell line showed a significantly-increased hyperphosphorylated tau expression compared with others (Figure 4A,B and Figure S3E).

Next, we measured AT8 expression in neurons using immunocytochemical assay since tau proteins normally exist in axons as soluble forms and are accumulated in soma and dendrites when they become highly-or hyper-phosphorylated in the case of neurodegenerative disease progression [28]. As expected, we observed high expression levels of phosphorylated tau at AT8 epitope in *MAPT P301L* cell line. By contrast, no significant differences were observed between bvFTD-iPSNs and the normal control iPSC-derived neurons (Figure 4C,D).

### 2.6. TDP-43 and FUS Expression in the bvFTD iPSC-Derived Neurons

In the absence of any stimuli, FUS and TDP-43 proteins play important roles in shuttling other players between the nucleus and cytoplasm and are normally localized in the nucleus [29]. According to the previous reports, FTD patients exhibit some abnormal localization of FUS and TDP-43 protein expression in their post-mortem brain image analysis [29]. The major feature of mis-localization is cytoplasmic aggregates that are accumulated around the nucleus since these proteins fail to translocate through the nuclear envelope, leading to the loss of function in the nucleus and the gain of toxic effects in the cytoplasm [4,26,30]. It is also known that downregulated protein expression can cause protein pathologies [31]. Thus, we investigated the expression pattern of these proteins in our bvFTD iPSC-derived neurons.

**Figure 5:**
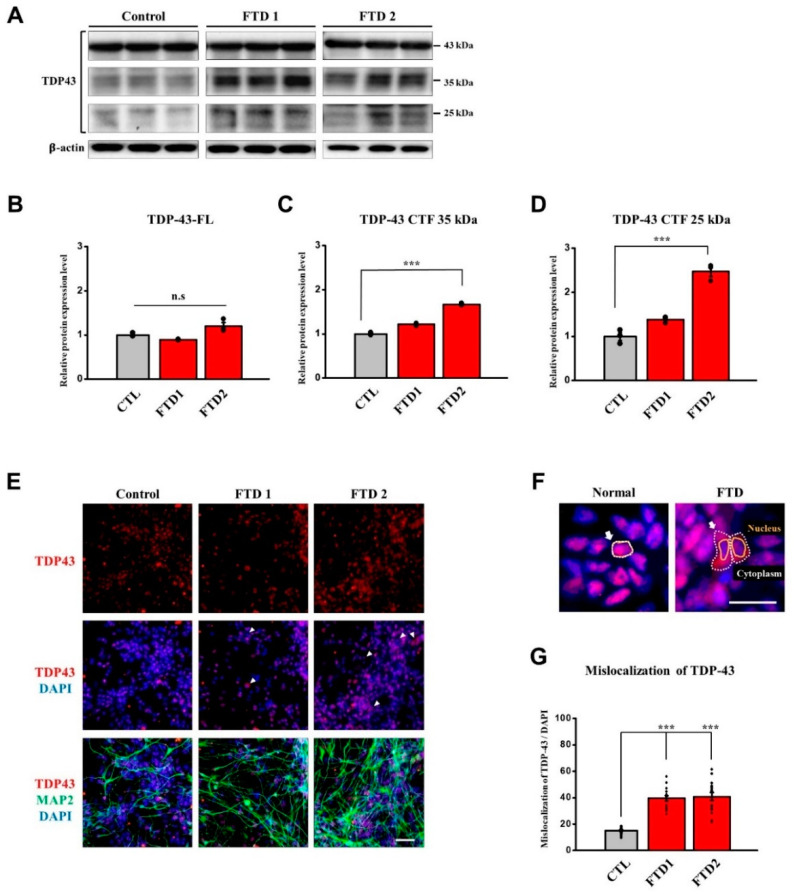
Abnormal expression patterns of TDP-43 protein in the bvFTD iPSC-derived neurons. (**A**) Immunoblot assay showing TDP-43 expression pattern full-length (FL): 43 kDa, fragments: 35 kDa, 25 kDa. β-actin was used as a loading control. Note that blot images indicated by dividing lines were chosen from different immunoblots due to the large size of samples. Protein quantifications were conducted under the same exposure time. Whole blot images are included in Supplementary Figure S4A–C. (**B**) Quantification of immunoblot assay showing the full length of TDP-43 protein expression. (**C**) Quantification of immunoblot assay showing the 35 kDa fragments of TDP-43 protein expression. (**D**) Quantification of immunoblot assay showing that 25 kDa fragments of TDP-43 protein expression levels were significantly increased in bvFTD2iPSC-derived neurons (*n* = 3) (*: vs. normal control) * *p* < 0.05, ** *p* < 0.01, and *** *p* < 0.001, one-way ANOVA. (**E**) Immunocytochemical assay showing the expression of TDP-43 (red) and MAP2 (green), which were counter-stained with DAPI (blue), in the iPSC-derived neurons at three weeks of neuronal differentiation. Scale bar = 50 μm. (**F**) Simple description of mis-localized TDP-43 expression. Scale bar: 100 μm. (**G**) Quantification of immunocytochemical assay using the translocation module in the MetaXpress^®^ 6 program. Note that mis-localized TDP-43 expression against the nucleus was increased in two bvFTD iPSC-derived neurons (*n* = 16) (*: vs. normal control) * *p* < 0.05, ** *p* < 0.01, and *** *p* < 0.001, one-way ANOVA.

First, we quantified the different molecular sizes of TDP-43 proteins since previous studies showed that TDP-43 proteins have specific caspase-3 cleavage consensus sites [32] that will produce 35 kDa and 25 kDa fragments when they become cleaved by the active caspase-3. Therefore, we examined the expression patterns of both 35 kDa and 25 kDa fragments using immunoblot assay (Figure 5A, Figure S4A–C).

Interestingly, no significant change of the full-length TDP-43 expression was observed, compared with the normal control. However, we observed that only bvFTD2-iPSN was significantly increased in both 35 kDa and 25 kDa TDP-43 fragments, compared with the normal control (Figure 5B–D).

Next, we targeted the cytoplasmic aggregates accumulated around the nuclei (as marked in Figure 5F) and measured this mis-localized TDP-43 protein expression using the translocation module in the program. As a result, we observed that the mis-localized TDP-43 protein expression pattern was significantly increased in the two bvFTD-iPSNs, compared with the normal control (Figure 5E,G). These results suggest that pathological phenotypes of bvFTD2-iPSN could be related with abnormal expression pattern of TDP-43.

**Figure 6:**
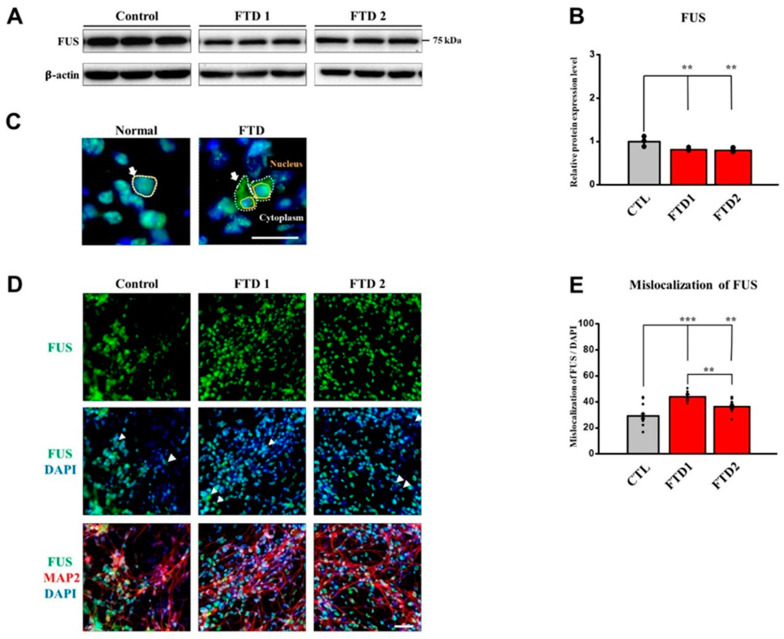
Abnormal expression patterns of FUS proteins in the bvFTD iPSC-derived neurons. (**A**) Immunoblot assay showing FUS expression. β-actin was used as a loading control. Note that blot images indicated by dividing lines were chosen from different immunoblots due to the large size of samples. Protein quantifications were conducted under the same exposure time. Whole blot images are provided in Supplementary Figure S4D. (**B**) Quantification of immunoblot assay showing that total FUS expression levels were decreased in the two bvFTD iPSNs (*n* = 3) (*: vs. normal control) * *p* < 0.05, ** *p* < 0.01, and *** *p* < 0.001, one-way ANOVA. (**C**) Simple description of mis-localized FUS expression. Scale bar: 100 μm. (**D**) Immunocytochemical assay showing the expression of FUS (green) and MAP2 (red), which were counter-stained with DAPI (blue), in the iPSC-derived neurons at three weeks of neuronal differentiation. Scale bar: 50 μm. (**E**) Quantification of immunocytochemical assay using the translocation module in the MetaXpress^®^ 6 program. Note that mis-localized FUS expression against the nucleus was increased in the two bvFTD iPSCNs, bvFTD1 (*p* < 0.0001) and bvFTD2 (*p* = 0.0029) (*n* = 16) (*: vs. control) * *p* < 0.05, ** *p* < 0.01, and *** *p* < 0.001, one-way ANOVA.

Although total FUS expression was decreased in all bvFTD-iPSNs compared with the normal control (Figure 6A,B and Figure S4D) we also observed increased levels of mis-localized FUS expression against DAPI (nucleus) in the two bvFTD-iPSNs by immunocytochemical analysis, compared with the normal control (Figure 6C–E). Since bvFTD1-iPSN showed significantly increased mis-localized expression of FUS compared with the bvFTD2-iPSNs, our findings suggest that bvFTD1-iPSN is more likely to be related with FUS dysfunction.

## 3. Discussion

Next to Alzheimer’s disease, FTD is the second-most-common cause of neurodegenerative dementia, showing behavioral problems typically in people younger than 65 years of age [1,2]. FTD exhibits atrophy mainly in the frontal and temporal lobes, leading to FTLD associated with a wide range of heterogeneous pathologies. In this study, we applied iPSC technology to model sporadic bvFTD, hoping this approach would aid in the elucidation of disease mechanisms as well as drug discoveries for FTD in the future. To this end, we differentiated one normal control and two sporadic bvFTD-patient-derived iPSCs, along with one positive control iPSC-line-carrying *MAPT P301L* mutation into post-mitotic neurons.

A 74 year-old normal control and two young bvFTD patients in their thirties were chosen for this study. It is highly challenging to define the ‘normal control’ in the field of modeling neurodegenerative diseases using iPSC technology, especially when one needs to model sporadic diseases. The isogenic iPSC line would be the most ideal choice as a normal control against the patient-derived iPSC line if the target gene was defined. However, in sporadic cases with unknown genetic background, we have no option but to choose non-demented subjects who have lived long, healthy, and cognitively-normal lives.

We first confirmed that our two bvFTD-iPSNs did not produce abnormal amyloid beta peptides compared with the control, in which amyloid PET scans were also negative. Next, we investigated the FTD-related phenotypes. Immunoblot analyses indicated that that bvFTD-iPSNs did not generate hyperphosphorylated tau (AT8 epitope) compared with the normal control and *MAPT* P301L-iPSNs. However, increased level of TDP-43 c-terminal fragments in bvFTD2-iPSN and decreased total FUS expression levels in both bvFTD-iPSNs were observed, compared with the normal control. To further quantify images, we employed ImageXpress^®^ Micro confocal to acquire images and subsequently Metaxpress^®^6 program to quantify the protein expression levels. These high-content-screening (HCS) modules allow non-biased image analyses.

First of all, we observed that both of our bvFTD-iPSNs did not significantly express hyperphosphorylated tau compared with controls, suggesting that our bvFTD iPSC lines were unrelated to FTLD-tau pathology.

Secondly, although we observed mis-localized expression patterns of FUS and TDP-43 proteins expression around the nuclei in both bvFTD-iPSNs, it is less likely that young bvFTD patients have both FUS and TDP-43 as underlying pathology. Since bvFTD1-iPSN showed significantly increased mis-localized expression of FUS compared with the bvFTD2-iPSNs and only bvFTD2-iPSN showed increased TDP-43 fragments, we speculate that bvFTD1-iPSNs might be related to the FUS malfunction while bvFTD2-iPSN might be related to TDP-43 fragmentation. However, these assumptions need to be verified using more cases. In this case, *MAPT P301L*-iPSN was not included for comparison as it is not related to the FUS and TDP43 pathology. Therefore, further studies using more bvFTD patients with defined familial FTD-iPSC lines for FUS and TDP43 pathology such as *PGRN* mutation will be useful to verify our results.

Thirdly and most importantly, we observed that active caspase-3 expression can be a useful marker to demonstrate the definite brain atrophy in bvFTD-specific iPSC lines. Our bvFTD-iPSNs in the presence or absence of STS treatment expressed high levels of cell-death-related proteins, such as Bcl-2, Bax, and cytochrome C, whereas active caspase-3 expression was induced only after STS treatment.

## 4. Materials and Methods

### 4.1. Participants

Two sporadic bvFTD patients and one normal control were recruited from Samsung Medical Center, Seoul, Korea from April 2015 to October 2016. Both patients met the diagnostic criteria for bvFTD proposed by Rascovsky et al. [3]. We performed detailed neuropsychological tests [33,34] to evaluate which cognitive domain was impaired, and brain MRI to evaluate atrophy pattern. To evaluate whether the participants had amyloid deposition in the brain, we performed ^18^F-florbetaben PET for all participants. To evaluate for paired helical filament tau deposition in the brain, the two bvFTD patients additionally underwent ^18^F-flortaucipir PET at Gangnam Severance Hospital. To search for mutations that can cause bvFTD, we performed WES for the two bvFTD patients. The institutional review board of the Samsung Medical Center and CHA University approved the study protocol. Informed written consent was obtained from each participant and their next to the kin [35].

### 4.2. Whole Exome Sequencing and Data Analysis

Genomic DNA was extracted from peripheral blood leukocytes, and exome sequencing was performed using SureSelect Human All Exon V6 (Agilent Technologies, Santa Clara, CA, USA) and Illumina HiSeq2500 platform (Illumina Inc, San Diego, CA, USA). We screened known variants for FTD-related genes, including *ALS2, ANG, CHMP2B, CHRNA4, DAO, DCTN1, FIG4, FUS, GRN, HNRNPA1, HNRNPA2B1, MAPT, MATR3, OPTN, PRNP, SETX, SIGMAR1, SOD1, SPG11, SQSTM1, TAF15, TARDBP, TBK1, TREM2, UBQLN2, VAPB, VCP, AARS2, ABCD1, APP, ARSA, CSF1R, DARS2, EIF2B1, EIF2B2, EIF2B3, EIF2B4, EIF2B5, GALC, GBA, GLA, NOTCH3, PSEN1, PSEN2, SNCB*, and *TYROBP*. Candidate variants were classified according to the standards and guidelines of the American College of Medical Genetics and Genomics and the Association for Molecular Pathology [36].

### 4.3. Generation of iPSCs Using Sendai Virus Vector and iPSC Culture

The iPSC lines were generated and maintained by using the same method reported previously [23].

### 4.4. Detection of Residual Sendai Virus Expression and Mycoplasma Detection PCR

The iPSC lines were analyzed for the presence of residual Sendai virus (SeVdp) expression and the contamination by mycoplasma using the same methods reported previously [23].

### 4.5. Neural Induction

Neural precursor cells (NPCs) were induced and cultured using the same methods reported previously [23].

### 4.6. Post-Mitotic Neuronal Differentiation

Neural precursor cells were differentiated to post-mitotic neurons for 3 weeks with neurobasal-A medium (Thermo Fisher Scientific, Waltham, MA, USA) containing 1% B27 supplement without vitamin A (Thermo Fisher Scientific), 1% antibiotic-antimycotic solutions, 10 ng/mL brain-derived neurotrophic factor (BDNF), 10 ng/mL glial cell-derived neurotrophic factor, 200 mM ascorbic acid (Sigma, St. Louis, MO, USA), and 1mg db-cAMP (PeproTech, Seoul, Korea) on PLO/laminin-coated dishes [23].

### 4.7. Cellular Stress-Induced Caspase-3 Activation Assay

Neurons differentiated for 3 weeks were treated with 100nM of staurosporine (S5921, Sigma) for 24 h prior to sample preparation for analysis [13].

### 4.8. Immunocytochemistry

All neurons were prepared by the methods described in a previous report [23]. The following primary antibodies were used: anti-OCT4 (1:200, Santa Cruz Biotechnology, Santa Cruz, CA, USA), anti-SOX2 (1:200, Millipore, Burlington, MA, USA), anti-NANOG (1:200, R&D Systems), anti-SSEA-4 (1:100, Developmental Studies Hybridoma Bank, Iowa City, IA, USA), anti-TRA-1-81 (1:100, Thermo Fisher Scientific), Tuj1 anti-tubulin beta III isoform (1:200, Millipore), Tuj1 anti-tubulin beta III (1:200, Abcam, Cambridge, UK), anti-SMA (1:100, DAKO, Santa Clara, CA, USA), anti-AFP (1:100, DAKO), anti-Nestin (1:200, R&D Systems), anti-Musashi (1:200, Millipore), anti-MAP2 (1:200,Millipore), anti-TDP43 (1:200, Proteintech, Rosemont, IL, USA), anti-FUS/TLS (1:200, Santa Cruz Biotechnology), *p*-Tau (AT8), and anti-Caspase-3 (1:100, Millipore).

### 4.9. Image Acquisition and Quantifications

All the images of neurons for analyzing TDP-43, FUS, and *p*-Tau expression were acquired by using a high content screening (HCS) confocal microscopy (ImageXpress^TM^ Micro Confocal, Molecular Devices, San Jose, CA, USA) from 16 different sites of each specimen at 20× magnification. Moreover, images for analyzing caspase-3 expression were acquired from 36 different sites of each specimen at 60× magnification. The neurite outgrowth module from Metaxpress^®^6 program (Molecular Devices) was used for quantifying the intensity of *p*-Tau expression from neurons and the translocation module was used for quantifying the mis-localization of TDP-43 and FUS expression. The colocalization module was used for quantifying the cleaved caspase-3 expressed in neurons. To ensure unchanged image quantifications, all analyses were performed using the same set of parameters in each module. All images were normalized using the same number of nuclei.

### 4.10. Western Blot Analysis

Cultured neurons were harvested at 3 weeks of differentiation as cell pellets. Protein samples were prepared and assayed by the method described previously [23]. The following primary antibodies were used—anti-TDP43 (1:200, Proteintech), anti-FUS/TLS (1:200, Santa Cruz Biotechnology), Tau5 anti-tau (1:1000, Thermo Fisher Scientific), AT8 anti-p-tau (1:1000, Thermo Fisher Scientific), and anti-caspase-3 (1:200, Millipore).

### 4.11. Extracellular Aβ ELISA

Conditioned media (CM) were collected from cultured neuronal cells (2 × 10^5^) at 48 h after the last medium change from 3 weeks of differentiation onwards. Aβ40 and Aβ42 levels were measured using the human Aβ40 and Aβ42 ELISA kit according to the manufacture’s protocol (IBL, Minneapolis, MN, USA). ELISA plate reader (Multiskanä FC Microplate Photometer, Thermo Fisher Scientific) was used to quantify Aβ40 and Aβ42 levels.

### 4.12. Statistical Analysis

All statistical analyses were performed using one-way analysis of variance (ANOVA) followed by a Fisher’s LSD (least significant difference) using the Statistical Analysis System (Enterprise 4.1, SAS Korea, Seoul, Korea). Significance was accepted at the 95% probability level. Data in graphs are presented as the mean ± SEM (standard error of the mean). *p*-value < 0.05 (*), *p*-value < 0.01 (**), and *p*-value < 0.001 (***).

### 4.13. Ethics Approval and Consent to Participate

The institutional review board of the Samsung Medical Center and CHA University approved the study protocol (1044308-201612-BR-031-05). Informed written consent was obtained from each participant and their next to the kin [35].

## 5. Conclusions

Taken together, we demonstrated the disease modeling of a sporadic form of bvFTD using iPSC technology and showed the expression of cell-death-related proteins. In particular, active caspase-3 expression following STS treatment can be a useful biomarker to represent FTD pathology.

## Supplementary Materials

The following are available online at https://www.mdpi.com/1422-0067/21/15/5319/s1, Figure S1: Neuronal differentiation of bvFTD-patient-derived iPSCs. Figure S2: Amyloid beta ELISA of patient iPSC-derived neurons. Figure S3: Original immunoblot images in Figure 3A and Figure 4A. Figure S4: Original immunoblot images in Figure 5A and Figure 6A.
