# Supplementary figures and images for: Modeling of Frontotemporal Dementia Using iPSC Technology
Source: Int J Mol Sci. 2020 Jul 27;21(15):5319. doi: 10.3390/ijms21155319 (PMC7432206; doi:10.3390/ijms21155319)

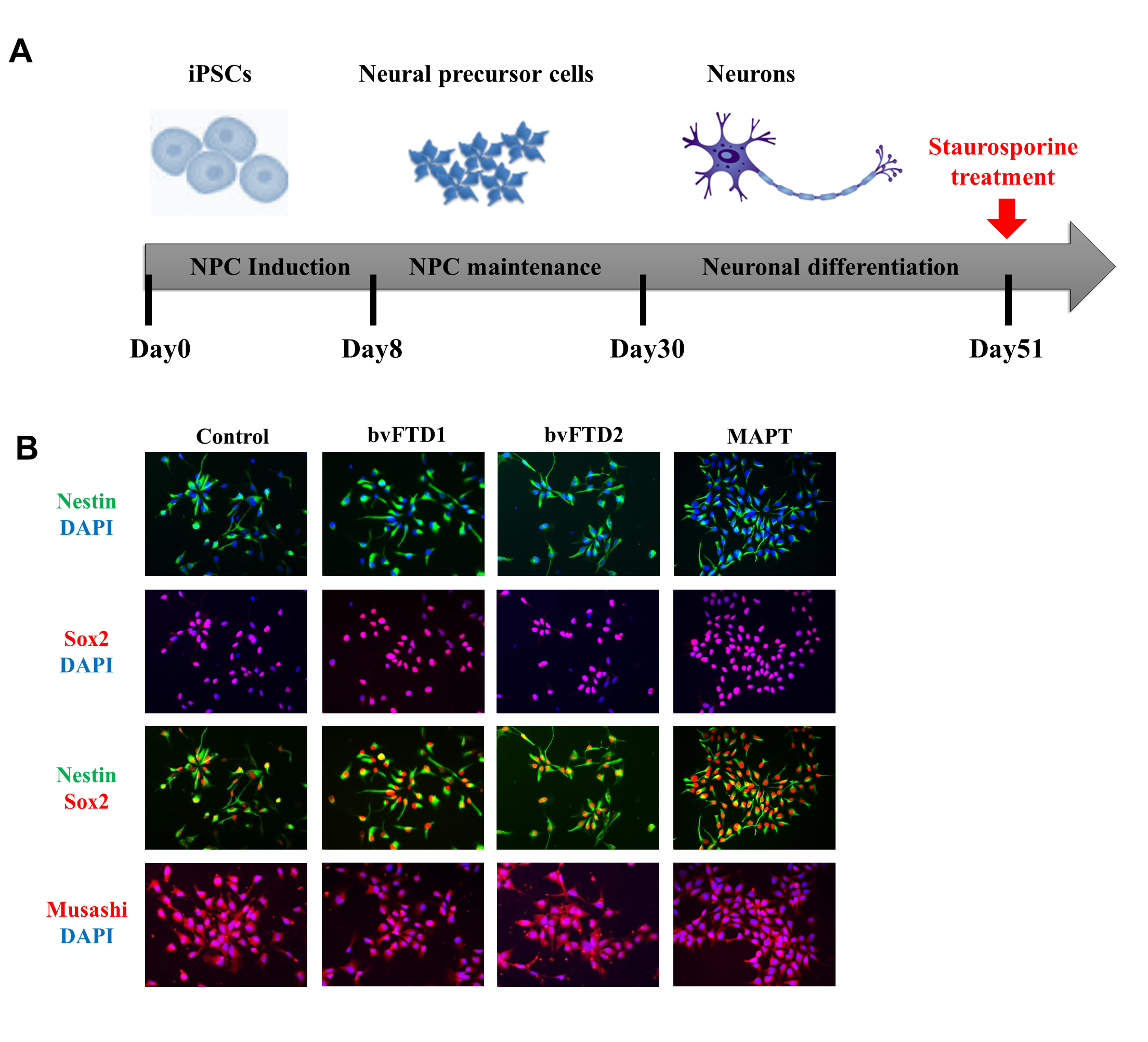

Supplement: Supplementary file 1 [file ijms-21-05319-s001.zip › Figure S1.tif]

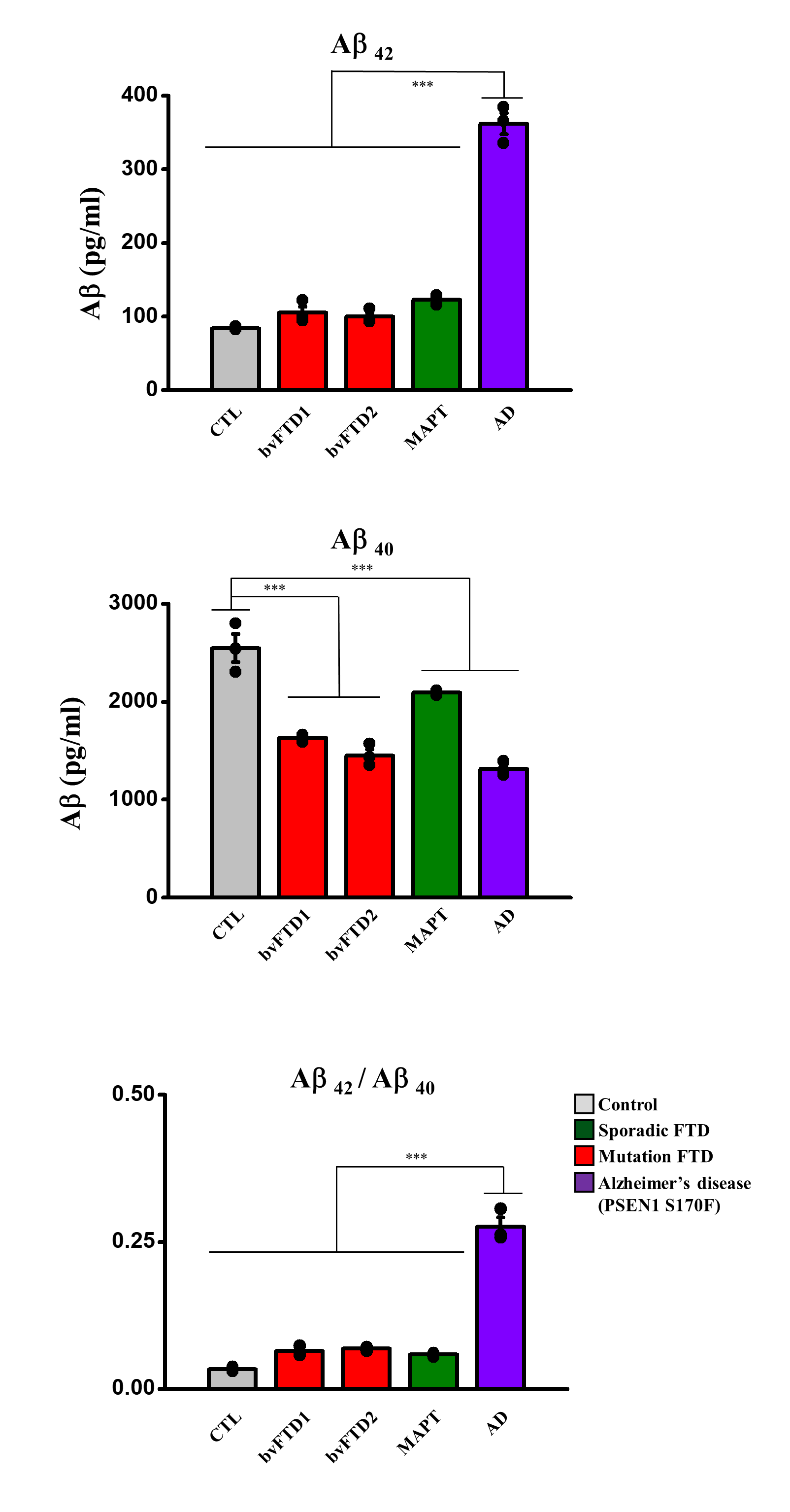

Supplement: Supplementary file 1 [file ijms-21-05319-s001.zip › Figure S2.tif]

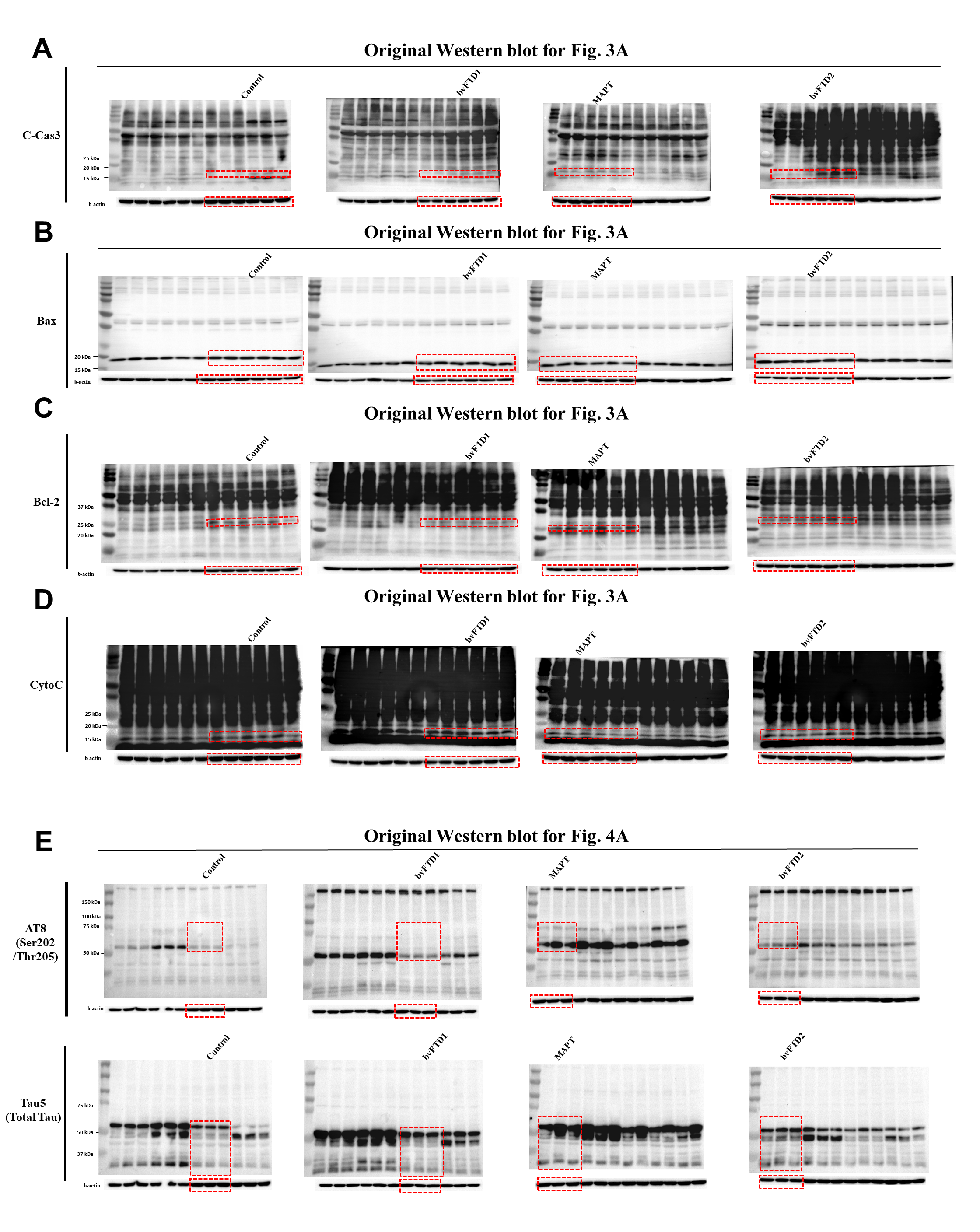

Supplement: Supplementary file 1 [file ijms-21-05319-s001.zip › Figure S3.tif]

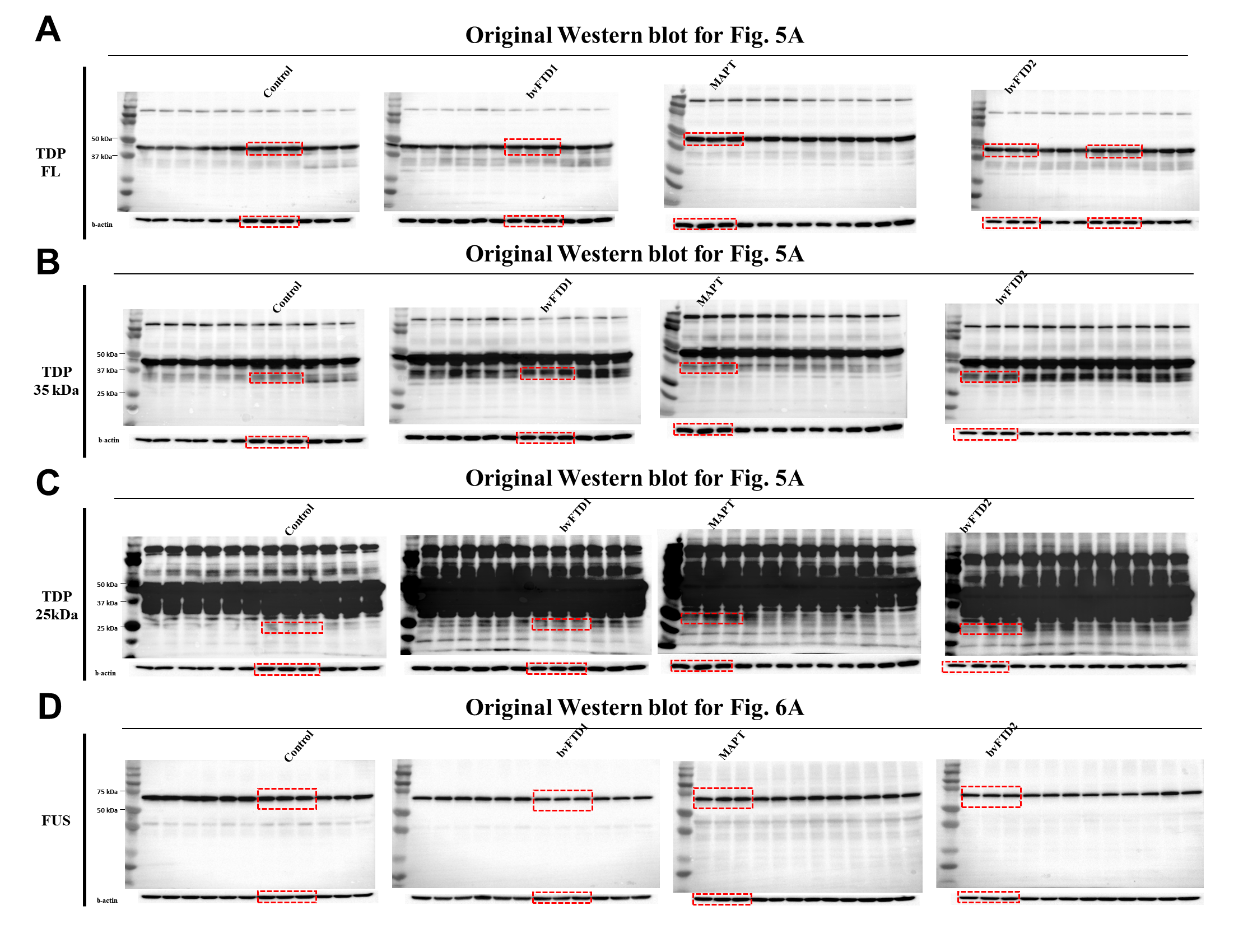

Supplement: Supplementary file 1 [file ijms-21-05319-s001.zip › Figure S4.tif]
